# Changes in Resources and Volunteering: A Longitudinal Study of Active Engagement Among Older Europeans

**DOI:** 10.1093/geront/gnae049

**Published:** 2024-05-20

**Authors:** Hans Hämäläinen, Antti O Tanskanen, Bruno Arpino, Liili Abuladze, Aïda Solé-Auró, Mirkka Danielsbacka

**Affiliations:** Department of Social Research, University of Turku, Turku, Varsinais-Suomi, Finland; INVEST Research Flagship Centre, University of Turku, Turku, Varsinais-Suomi, Finland; Department of Social Research, University of Turku, Turku, Varsinais-Suomi, Finland; Population Research Institute, Väestöliitto, Helsinki, Uusimaa, Finland; Department of Statistical Science and Department of Philosophy, Sociology, Education and Applied Psychology, University of Padua, Padua, Italy; Population Research Institute, Väestöliitto, Helsinki, Uusimaa, Finland; Estonian Institute for Population Studies, School of Governance, Law and Society, Tallinn University, Tallinn, Harju County, Estonia; Department of Political and Social Sciences, DemoSoc Research Group, Pompeu Fabra University, Barcelona, Catalonia, Spain; INVEST Research Flagship Centre, University of Turku, Turku, Varsinais-Suomi, Finland; Population Research Institute, Väestöliitto, Helsinki, Uusimaa, Finland

**Keywords:** Active aging, Engagement, Europe, Panel study

## Abstract

**Background and Objectives:**

Volunteering is an important dimension of successful aging. Although prior studies have found that personal resources such as health and financial situations are associated with volunteering, there is a lack of research exploring the relationship between resource changes and volunteering. Here, researchers investigated whether changes in individuals’ resources were associated with volunteer engagement among older Europeans.

**Research Design and Methods:**

Using data from the Survey of Health, Aging, and Retirement in Europe, collected in 5 waves between 2011 and 2020 across 19 countries (57 410 observations from 17 498 individuals aged 50 and older), researchers employed asymmetric fixed-effect ordinal regression models to investigate whether positive or negative resource changes were associated with the frequency of volunteering over time. Researchers used 3 resource indicators: health, financial condition, and time availability (measured by engagement in paid work, grandparenting, and family care).

**Results:**

Health deterioration and worsening financial condition were associated with a decreased frequency of volunteering. A transition out of weekly paid work and beginning to provide weekly grandchild care were both associated with an increased frequency of volunteering. Researchers did not detect any further significant effects of resource changes on volunteering.

**Discussion and Implications:**

Researchers study revealed asymmetrical associations between changes in resources and volunteering, providing new insights into their interplay. The results deepen researcher's understanding of successful aging by emphasizing the need to consider the dynamics of all resources that either facilitate or hinder active engagements among older adults.

## Background and Objectives

Due to the aging population structures and increasing longevity in Western countries, questions related to the aging of older adults have become a burning issue. Beyond individual attributes, such as good health, the quality of aging is argued to depend on active engagement in life (Morawski et al., 2022; Rowe & Kahn, 1997). Volunteering, defined as an unpaid activity that benefits parties with whom individuals do not have a personal relationship, stands out as a prime example of such an activity (Hämäläinen et al., 2023). While volunteering is a productive activity typically benefiting the community and society at large, it may also be very rewarding for older adults, as it can provide a meaningful role in helping others, facilitate the maintenance of social relationships, and help to form new ones (Morrow-Howell, 2010). However, volunteering depends on resources that enable individuals to engage in activities, such as health, financial conditions, and available time (Hämäläinen et al., 2024; Wilson & Musick, 1997).

Although previous studies have shown that older adults with better resources, such as improved health, are more likely to volunteer than those with worse resources (Cheng et al., 2022; Principi et al., 2016), there is very limited research on how *changes* in resources are associated with volunteering among older adults, with a few exceptions (Eibich et al., 2022; Hämäläinen et al., 2024; Papa et al., 2019). Furthermore, no previous studies have examined whether both increasing and decreasing resources are associated with volunteering. Researchers investigated how positive and negative changes in various types of resources are associated with volunteer participation among older Europeans.

### Active Engagements From the Resource Perspective

The concept of successful aging argues that the challenges related to individual aging can be addressed by postponing diseases and disabilities, maintaining good cognitive and physical functional capacities, and lengthening the period of active participation in society (Rowe & Kahn, 1997, 2015). This concept seeks to move beyond the stereotype of old age as a passive and unproductive phase of life. It emphasizes that successful aging does not only mean the absence of health problems and maintenance of functional abilities, but it is their combination with active engagement in life that truly represents successful aging (Rowe & Kahn, 1997). The relationships between these components (i.e., health, functioning, and active engagement) are viewed somewhat hierarchically, as individuals’ ability to engage in activities is dependent on their health and functional capabilities (Baik et al., 2024; Rowe & Kahn, 1997), that is, adequate health and functional capabilities are prerequisites for active engagement.

Although a key premise of successful aging is to postpone barriers that prevent older adults from engaging in activities, research often overlooks the dynamics of those issues. For instance, despite the risks of facing health and functional problems are undoubtedly increasing with age (Rowe & Kahn, 1997), older adults may nevertheless experience improvements in these aspects. Consequently, the ability of older adults to engage in activities is not stagnant but can fluctuate over time. In this study, researchers did not assume that people who are experiencing restrictions in their abilities to engage, such as health problems, would not be able to volunteer at all, but rather explored whether improving or diminishing abilities are associated with the frequency of participation in volunteering among older adults.

While focusing on the important aspects of health and functioning, the concept of successful aging tends to ignore other factors that may affect the ability to volunteer. According to the resource theory, volunteering is facilitated by all the resources that empower individuals to engage in such activities (Principi et al., 2016; Wilson & Musick, 1997, 1998). In addition to health and functional ability, these resources refer to other enabling or restricting factors, such as financial condition and availability of time. Resource theory predicts that the amount of resources individuals have is positively associated with volunteer participation (Cheng et al., 2022; Wilson & Musick, 1997). In this study, researchers combined the concepts of successful aging and resource theory to examine engagement in volunteering from the perspective of individuals’ resources, which are defined as personal assets that can enable or restrict volunteering.

Prior empirical results show that health is a crucial resource that enables volunteering (Baik et al., 2024). Older adults’ physical health varies substantially, influencing their ability to engage in different activities (Principi et al., 2016); individuals with better health are more likely to volunteer than those who are not all that healthy (Choi, 2003; Hank, 2011). A study using longitudinal data collected from individuals aged 50 and older in 13 European countries found that a decline in health (measured as mobility limitations and depression) decreased the likelihood of volunteering (Papa et al., 2019). Financial resources also play an important role in empowering individuals to volunteer. Previous studies have found that those with higher incomes are more likely to volunteer than those with lower incomes (Choi, 2003; Principi et al., 2016). Although volunteering does not involve giving money, a more secure financial situation may reflect an individual’s ability to devote more time to nonpaid activities. Additionally, improved financial condition may enhance the ability to cover the costs related to participation in volunteering activities, such as traveling and equipment expenses, as well as reflect better access to information and technologies that facilitate volunteering (Arpino & Solé–Auró, 2023).

The ability to volunteer is also dependent on an individual’s time resources. As time is strictly limited, the question is how time is allocated between different activities. For instance, previous studies have indicated that retirees are more likely to volunteer than their employed counterparts (Hank & Erlinghagen, 2010; Mutchler et al., 2003). A recent investigation (Eibich et al., 2022) explored the association between retirement and volunteering further by employing an instrumental variable approach while utilizing longitudinal data from 12 European countries and the United States. The study found that retirement had a positive effect on the frequency of volunteering in all the countries included in the investigation. Similarly, an investigation employing panel fixed-effect regression and using longitudinal data from 19 European countries detected that the transition from employment to retirement was associated with an increased frequency of volunteering (Hämäläinen et al., 2024). Besides employment, engagement in other types of activities, especially those typically valued by people, such as the provision of care for family members, also limits the time available to be spent on volunteering (Baik et al., 2024; Morawski et al., 2022).

### The Present Study

In this study, researchers investigated how positive and negative changes in individuals’ resources are associated with volunteering among older Europeans. Researchers explored multiple types of resources: (i) overall health (measured by self-rated health), (ii) economic condition (measured by self-rated financial condition), and (iii) time availability (measured by engagement in other time-consuming activities, such as paid work, grandparenting, and family care). Based on the resource theory of volunteering, researchers assumed:

*Hypothesis 1*: A change to worse resources (i.e., deterioration of health and worsening financial conditions, increased engagement in paid work, grandparenting, and family care) is associated with a decreased frequency of volunteering.

*Hypothesis 2:* A change to better resources (i.e., improvement in health and financial conditions, decreased engagement in paid work, grandparenting, and family care) is associated with an increased frequency of volunteering.

## Research Design and Methods

### Data

Researchers used longitudinal data from the Survey of Health, Aging and Retirement in Europe (SHARE). SHARE collects data through computer-assisted personal interviews with people aged 50 and older (Börsch-Supan et al., 2013). In the present study, the sample included respondents from the fourth to eighth waves of SHARE, which were conducted every second year between 2011 and 2020 in 19 European countries: Austria, Belgium, Croatia, the Czech Republic, Denmark, Estonia, France, Germany, Greece, Hungary, Italy, Luxembourg, the Netherlands, Poland, Portugal, Slovenia, Spain, Sweden, and Switzerland. The first two waves of data were excluded, as the questions about volunteering were phrased differently than in the later waves. The third wave (SHARELIFE) was not included because of its retrospective life history focus and a different questionnaire. The seventh wave of data collection consisted of both SHARELIFE and a condensed regular questionnaire for those new to SHARELIFE (i.e., those who had not participated in Wave 3), whereas the regular questionnaire was directed to those who had participated in SHARELIFE earlier. However, the main variables of interest were included in both of those questionnaires (except variables measuring grandchild and family care), enabling us to utilize the seventh wave. The eighth wave was interrupted by the COVID-19 pandemic, and researchers used data collected before the outbreak of the pandemic. As a consequence of the interruption of Wave 8, the countries that entered SHARE in Wave 7 included only a few respondents who had participated in at least two waves; for that reason, those countries were excluded from the analyses. The final study sample included 57 410 observations from 17 498 individuals who participated in at least two study waves. The distribution of observations across the study samples is presented in Supplementary Table S1 in the Supplementary Material.

### Dependent Variable

In SHARE, respondents were asked whether they had engaged in volunteering in the past 12 months. Those who answered “yes” were asked to report the frequency of their participation. For analysis, these responses were combined into one ordinal volunteering variable ranging from 0 to 4, where 0 = No volunteering, 1 = Less than monthly, 2 = Almost monthly, 3 = Almost weekly, and 4 = Almost daily.

### Main Independent Variables

The main independent variables concern the following individual-level resources for volunteering: (i) health, (ii) finances, and (iii) time. For the analysis, researchers created dummy variables indicating the threshold between decent and scarce resources. In the questionnaire, respondents were asked to rate their health (self-rated health) using a 5-point scale: 1 = Excellent; 2 = Very good; 3 = Good; 4 = Fair; 5 = Poor, which was recoded into 0 = Good or better (“Excellent,” “Very good” or “Good”) and 1 = Fair at best (“Fair” or “Poor”). Respondents’ financial conditions were assessed by inquiring how easily their household made ends meet: 1 = Easily, 2 = Fairly easily, 3 = With some difficulty, and 4 = With great difficulty. The financial condition was recoded to 0 = No difficulties (“Easily” or “Fairly easily”) and 1 = Difficulties (“With some difficulties” or “With great difficulties”).

Time resources were evaluated based on respondents’ engagement in other time-consuming activities such as employment, grandparenting, and provision of family care. In the questionnaire, individuals were asked how many hours they usually did paid work per week. The variable was dichotomized as 0 = None and 1 = At least some work. For sensitivity purposes, this threshold was changed to 8 and 20 h of work per week, but this did not alter the results. Regarding grandchild care, respondents who had at least one grandchild were asked to report whether they had looked after the children of each of their own children without their parents being present, either during the time interval since the last interview (in follow-up waves) or during the preceding 12 months (in the wave during which a participant entered SHARE), and if so, how often, using a scale of 1 = Almost every day, 2 = Almost every week, 3 = Almost every month, and 4 = Less often. Researchers transformed the scale to represent the number of days of grandchild care per year (0, 6, 12, 52, and 365 days). After converting the scale, researchers summed up the days of care provided to all children of their offspring and then capped the value at a maximum of 365 days per year (approximately 1% in each wave had a greater value and were recoded to 365). Finally, researchers created a variable measuring whether a respondent had provided grandchild care on average for at least one day per week (0 = No; 1 = Yes). Those respondents who did not have grandchildren were included in the “No” category. Regarding family care, respondents were asked whether they regularly provided care (e.g., washing, getting out of bed, dressing) to someone living in the same household during the past 12 months (0 = No, 1 = Yes). It was clarified that “regularly” means providing care almost daily for at least 3 months.

### Control Variables

In the analyses, researchers controlled for respondents’ age and changes in marital status because prior studies have indicated that these may affect the frequency of volunteering (Bolano & Arpino, 2020; Butrica et al., 2009; Principi et al., 2016). Moreover, as previous studies have shown that volunteering rates vary across European countries and that the variation aligns with countries’ prosperity (Morawski et al., 2022) and the strength of the welfare state (Erlinghagen & Hank, 2006; Hank & Erlinghagen, 2010), researchers controlled for countries’ total social expenditures in the year of the interview. Data on social expenditures during the study waves at constant 2010 prices were drawn from Eurostat (2023), encompassing all public spending on social protection, including benefits related to old age, unemployment, sickness, family, and housing. The expenditures per country, averaged over the observation period, and number of observations are presented in Supplementary Table S2. Moreover, by employing panel fixed-effect regression models, researchers accounted for all time-invariant factors, including country-level time-constant variables, as discussed in the Section Statistical Analyses.

### Statistical Analyses

Researchers used within-person (or panel fixed-effects) models to investigate whether changes in individuals’ resources were associated with volunteer participation. Within-person models consider individual-specific changes and present variations in individual behavior over time. In within-person models, repeated measures (i.e., person-observations) are nested within responding individuals (Jokela et al., 2018). The participants serve as their own controls, and these models eliminate all time-invariant factors (Allison, 2009; Brüderl & Ludwig, 2015), meaning that factors that do not change over time are controlled for regardless of whether they are available in the data, such as sex, education, stable personality traits, many genetic factors, and other selection effects. For this reason, as mentioned earlier, researchers did not include country dummies (which would be collinear with the individual fixed effect) but controlled for a relevant time-variant country-level factor.

Standard fixed-effects methods assume that the effects of the variables are symmetric; that is, the effect of increasing a variable corresponds to decreasing that variable, but in the opposite direction (Allison, 2019). However, decreasing or increasing resources among older adults can have different effects on volunteering, implying that this association may be asymmetrical. To take this into account, researchers employed an asymmetric fixed-effects regression, an approach used by Uccheddu et al. (2019) that builds on a panel fixed-effects regression model. This allowed to differentiate the effects of changes between worse and better resources. Researchers defined two counter variables measuring changes in each resource variable over the study waves: one increases with each additional change into worse resources (e.g., transition into financial difficulties), and the other increases with each additional change into better resources (e.g., transition out of financial difficulties). To do this, researchers differentiated the scores of the dummy variables into positive and negative components, representing an increase or decrease in resources between the observed time points (0 = No change; 1 = Change). If the value was not observed (e.g., respondent’s first available observation), the score was set to zero because no change could have been observed. Finally, to account for the fact that after a given change is observed, individuals can be observed in the new condition a varying number of times, researchers calculated the sum scores of both positive and negative changes, resulting in two variables: one representing the accumulation of all positive changes and the other showing the accumulation of all negative changes over the study waves. These variables were treated continuously in the analyses. This operationalization allowed us to utilize within-person models and separately examine the effects of changes to better and worse resources on volunteering (Allison, 2019; Uccheddu et al., 2019).

To investigate the association between changes in individuals’ resources and volunteering, researchers executed fixed-effect ordinal logistic regression models using the *feologit* command in Stata (see Baetschmann et al., 2020). The results from the ordinal logistic models are presented as regression coefficients in the tables. Average marginal effects cannot be estimated for fixed-effects ordered logit models; instead, researchers illustrate the results in figures by showing the marginal effects (ME) at the average with 95% CIs, as calculated from the regression models using *logitmarg* postestimation command (Baetschmann et al., 2020). All statistical analyses were performed using Stata 18 software.

## Results

### Descriptive Statistics

Most respondents (92%) were born in the country of data collection when they entered the survey. The sex distribution of the sample was slightly unbalanced: 44% were men and 56% were women. Regarding educational achievements, 12% of the respondents had at most primary education, 14% had completed lower secondary education, 41% had completed upper secondary non-tertiary education, and 33% had at least lower tertiary education. Descriptive statistics for all time-variant variables included in the analyses are shown in Table 1. For descriptive purposes only, country-level statistics on volunteering and social expenditures are provided in Supplementary Table 2. Social expenditures are lowest in Eastern European countries and highest in Northern and Western European countries, while Southern European countries lie somewhere in between. Regarding volunteering, a similar trend was observed in the study sample, although the pattern was not as clear as that of social expenditures.

**Table 1.** Descriptive Statistics

Characteristic	%	Mean	Within-person *SD*	Min–Max
Age at interview		65.8	2.2	50–104
Marital status				
Married or registered	72.0			
Never married	6.0			
Divorced	10.0			
Widowed	12.0			
Self-rated health				
Fair at best	27.9			
Good or better	72.1			
Financial condition				
Difficulties	25.0			
No difficulties	75.0			
Weekly work				
Yes	28.2			
No	71.9			
Weekly grandchild care				
Yes	20.4			
No	79.6			
Family care				
Yes	6.1			
No	93.9			
Total social expenditures (k€)		9.6	0.3	1.84–18.38
Volunteering				
No volunteering	46.4			
Less than monthly	12.0			
Almost monthly	15.8			
Almost weekly	19.2			
Almost daily	6.7			

*Notes*: *n* = 57 410 person-observations from 17 498 unique individuals. *SD* = standard deviation.

According to the transition probabilities of volunteering, a significant share of the respondents remained in the same category over time; when changes occurred, there was more often a transition between categories close to each other than those further apart, or a change from volunteering to not volunteering at all (Supplementary Table 3). Descriptive statistics for the main independent variables that measured positive and negative changes in resources over the study waves are shown in Supplementary Table 4. For each measurement, most respondents did not experience a change during the study period; among those who experienced a change, the vast majority experienced it only once. It is worth noting that in order to encounter two changes in the same direction, individuals must have experienced a change in the opposite direction between those changes.

### Resource Changes and Volunteering

Table 2 presents the results of the fixed-effect ordinal regression model considering the association between resource changes and the frequency of volunteering. After controlling for time-invariant characteristics and other variables in the model, a change to worse health and a change to worse financial condition both had a negative effect on the frequency of volunteering. In contrast, transitioning out of weekly work and providing weekly grandparental childcare had a positive effect on the frequency of volunteering.

**Table 2.** Ordinal Logistic Regression: Associations Between Resource Change and Volunteering

Variable	Coef.	*SE*	95% CIs	
Resource change variables				
Change to worse health	−0.12**	**0.04**	**−0.19**	**−0.04**
Change to better health	**−**0.01	0.04	**−**0.08	0.08
Change to worse financial condition	**−0.13** ^**^	**0.04**	**−0.22**	**−0.04**
Change to better financial condition	0.05	0.04	**−**0.03	0.13
Transition to weekly work	0.07	0.07	**−**0.06	0.20
Transition out of weekly work	0.42***	**0.03**	**0.35**	**0.49**
Transition to weekly grandparenting	0.20***	**0.05**	**0.10**	**0.30**
Transition out of weekly grandparenting	**−**0.08	0.05	**−**0.18	0.02
Transition to family care	**−**0.10	0.07	**−**0.23	0.04
Transition out of family care	0.04	0.07	**−**0.09	0.18
Age at interview	−0.04***	**0.01**	**−0.05**	**−0.02**
Marital status				
Married or registered				
Never married	0.08	0.27	**−**0.45	0.62
Divorced	0.06	0.15	**−**0.24	0.36
Widowed	0.06	0.09	**−**0.11	0.24
Social expenditures	**−**0.05	0.04	**−**0.12	0.02

*Notes*: *n* = 57 410 person-observations from 17 498 unique individuals. CI = confidence interval; *SE* = standard error. Coefficients printed in bold are statistically significant (*p* < .05).

***p < .001. **p < .01. *p < .05.

**Figure 1.**
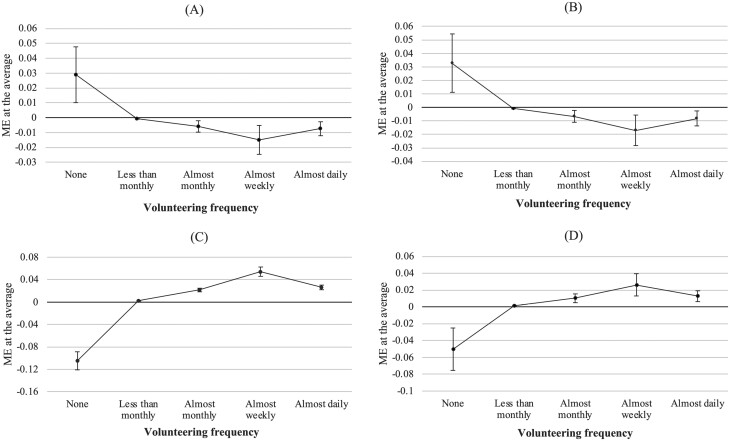
Marginal effects (ME) of resource changes on volunteering with 95% confidence intervals. (**A**) Change to worse health; (**B**) Change to worse financial condition; (**C**) Transition out of weekly work; (**D**) Transition to weekly grandparenting.

To better interpret the results, Figure 1 shows the marginal effects (ME) at the average on the probability of each category of the outcome (volunteering frequency) as calculated from the regression model (all marginal effects are shown in Table 3). Both a change to worse health (Figure 1A) and a change to a worse financial condition (Figure 1B) increased the likelihood of falling into the “no volunteering” category and decreased the probability of falling into any other outcome category (i.e., different frequency levels of volunteering). The magnitude of these statistically significant marginal effects was approximately 3% points for nonvolunteering and notably lower for all other levels of volunteering. Transitioning out of weekly work (Figure 1C) and beginning to provide weekly grandparental childcare (Figure 1D) were both associated with a decreased probability of not volunteering and an increased probability of all levels of volunteering. Here, the magnitude of the statistically significant effects is also substantially sizable, but mainly in the case of the effects on “no volunteering” (MEs of −10% and −5% points).

**Table 3.** Marginal Effects at the Average Calculated From the Fixed-Effects Ordered Logit Model

	Volunteering frequency				
Variable	No volunteering	Less than monthly	Almost monthly	Almost weekly	Almost daily
Change to worse health	0.029**	−0.001**	−0.006**	−0.015**	−0.007**
	(0.01)	(0.0002)	(0.002)	(0.005)	(0.002)
Change to better health	0.0002	**−**0.00001	**−**0.00005	**−**0.0001	**−**0.0001
	(0.011)	(0.0002)	(0.002)	(0.005)	(0.003)
Change to worse financial condition	0.033**	−0.001**	−0.007**	−0.017**	−0.008**
	(0.011)	(0.0003)	(0.002)	(0.006)	(0.003)
Change to better financial condition	**−**0.012	0.0003	0.003	0.006	0.003
	(0.01)	(0.0002)	(0.002)	(0.005)	(0.003)
Transition to weekly work	**−**0.017	0.0004	0.004	0.009	0.004
	(0.017)	(0.0004)	(0.003)	(0.009)	(0.004)
Transition out of weekly work	−0.105***	0.002***	0.022***	0.054***	0.026***
	(0.008)	(0.0002)	(0.002)	(0.004)	(0.002)
Transition to weekly grandparenting	−0.05***	0.001***	0.01***	0.026***	0.013***
	(0.013)	(0.0003)	(0.003)	(0.007)	(0.003)
Transition out of weekly grandparenting	0.021	**−**0.0005	**−**0.004	**−**0.011	**−**0.005
	(0.013)	(0.0003)	(0.003)	(0.007)	(0.003)
Transition to family care	0.024	**−**0.001	**−**0.005	**−**0.012	**−**0.006
	(0.017)	(0.0004)	(0.003)	(0.009)	(0.004)
Transition out of family care	**−**0.011	0.0002	0.002	0.006	0.003
	(0.017)	(0.0004)	(0.004)	(0.009)	(0.004)

*Notes*: Standard errors in parentheses. Coefficients printed in bold are statistically significant (*p* < .05).

***p < .001. **p < .01. *p < .05.

## Discussion and Implications

Previous studies have highlighted volunteering as one of the key dimensions of successful aging (Rowe & Kahn, 1997, 2015), and ascertained that individuals’ resources, such as good health and a sound financial position, are associated with their participation in volunteering (Cheng et al., 2022; Principi et al., 2016). However, only a few studies have examined how changes in resources among older adults, such as a transition from employment to retirement (Eibich et al., 2022; Hämäläinen et al., 2024) or decline in health (Papa et al., 2019), are associated with volunteering. To the best of researchers’ knowledge, no study has examined whether both increasing and decreasing resources are linked to the frequency of volunteering. Researchers aimed to fill this gap in the literature by investigating whether positive and negative changes in individual resources are associated with volunteering among older Europeans.

Researchers’ results revealed that the associations between resource changes (i.e., health, financial condition, and time) and the frequency of volunteering are asymmetrical; that is, increases and decreases in resources do not have equivalent effects on volunteering. Consistent with a previous study showing that a deterioration in the health of older adults is associated with less volunteering (Papa et al., 2019), researchers found that a change to worse health was associated with decreased volunteering. However, adding to prior knowledge, researchers’ investigation also indicated that improved health did not have a statistically significant effect on volunteering. Researchers detected a similar asymmetrical effect regarding financial condition: a deterioration in financial condition had a negative effect on volunteering, whereas an improvement in financial condition was not correlated with volunteering. Similarly, prior investigations have shown that individuals with higher incomes are more likely to volunteer than those with lower incomes (Choi, 2003; Principi et al., 2016), although this study is the first to explore how changes in financial conditions are associated with volunteering over time.

In addition to health and financial conditions, researchers investigated whether changes in time resources (measured by engagement in paid work, grandparenting, and family care) were associated with volunteering. Consistent with the previous studies detecting a positive association between retirement and an increased frequency of volunteering (Eibich et al., 2022; Hämäläinen et al., 2024), researchers found that transitioning out of weekly work had a positive effect on volunteering. However, focusing on weekly work instead of retirement allowed us to investigate whether spending more time on work also correlates with changes in volunteering, although researchers did not detect an association between increased working hours and volunteering. Besides consuming limited time resources, employment is also an important type of active engagement, and volunteering might function as a substitute for paid work; that is, older adults may compensate for the absence of work by engaging in volunteering (Chambré, 1984; Eibich et al., 2022). In contrast, providing weekly grandchild care had a positive effect on volunteering. It is possible that weekly grandparenting is a sign of highly active older adults who are more likely to participate in multiple activities simultaneously (Hämäläinen et al., 2023; Tanskanen et al., 2022). Researchers results are congruent with those of a previous study finding that grandparental childcare was associated with increased engagement in leisure activities (Ates et al., 2022), suggesting that grandparenting may lead to a cumulation of activities. Regarding the provision of family care, however, researchers did not detect any statistically significant association with volunteering, which could be related to the fact that family caregiving could be more demanding for older adults than grandchild care. In addition, this finding may be at least partly due to the relatively small number of family caregivers in the study sample, meaning that there was a lack of statistical power.

### Strengths and Limitations

This study is the first to investigate the asymmetrical effect of resource changes on volunteering in individuals’ lives. The investigation was based on extensive SHARE data, in which the same individuals were interviewed multiple times, making it possible to study changes in resources and participation in volunteering over time. To take full advantage of the panel data, researchers ran within-person regression models that focused on individuals’ varying behaviors over time and removed all the time-invariant factors, including country-level time-constant variables. Employing an asymmetric within-person approach enabled to draw more causal interpretations of the associations between positive and negative resource changes and volunteering.

While the data enabled researchers’ rigorous methodological approach, selective panel attrition is a common condition in panel surveys in which the same individuals are interviewed repeatedly. In the present study, selective panel attrition may have been present if older adults who volunteered frequently were also more likely to participate in follow-up surveys, or if, for instance, those experiencing negative resource changes were more likely to drop out. In addition, as a consequence of using fixed-effects modeling, respondents who did not experience any change in the frequency of volunteering across the study waves were excluded from the analytical sample by design. This may limit the generalizability of researchers results, especially for individuals whose volunteering habits remained constant throughout the study waves. Although researchers’ approach enabled to consider the asymmetry of the associations between resources and volunteering, the downside is that the resource change variables included only one threshold (e.g., capturing a change from no financial difficulties to having financial difficulties, and vice versa). This is likely to underestimate the effect of change on volunteering and resulting in small effect sizes. For instance, a change from sound financial conditions to severe financial difficulty may have a greater effect. Studies should explore the effects of resource changes on volunteering and consider the extent of the change.

Furthermore, researchers focused on changes within individuals’ lives and did not explore the possible differences between countries (or other time-constant factors). Because the use and adequacy of personal resources for volunteering may be related to a given societal context, future studies should investigate whether the association between resource changes and volunteering varies between countries. For instance, scarce public spending on social protection indicates that, in general, people are more dependent on their families (Saraceno & Keck, 2010), which may lead individuals to prioritize spending their resources for the benefit of their family rather than using them for others, such as volunteering, thus diluting the effects of resource changes on volunteering.

Finally, researchers’ dependent and explanatory variables are all self-reported measures, which may be affected by measurement errors. If these errors are not random, conditionally on the observed controls, researchers estimate may be biased. Future studies may use alternative types of data, such as time use data to measure volunteering and engagement in other activities.

## Conclusions

As population structures age and longevity increases, the question of *how* older adults age has become an important issue. Successful aging is argued to be grounded in the combination of three main components: good health, functional abilities, and active engagement in life (Rowe & Kahn, 1997). Congruent with the concept of successful aging, which focuses on the importance of avoiding health and functional issues (Rowe & Kahn, 1997, 2015), researchers found that health deterioration led to a decrease in volunteering. Additionally, researchers’ findings revealed that avoiding financial difficulties and devoting less time to paid employment were associated with increased volunteer participation. Moreover, grandparenting was also linked to more frequent volunteering, which suggests that one type of activity can also promote engagement in another. Thus, researchers’ result indicate that successful aging may not be comprised of only the components of health, functional abilities, and active engagement, but also of other factors that enable participation in activities.

## Supplementary Material

gnae049_suppl_Supplementary_Materials

## Data Availability

This study utilizes data from the Survey of Health, Aging, and Retirement in Europe (SHARE). The data are available for scientific use and can be accessed through the SHARE Research Data Center (https://share-eric.eu/).
