# Supplementary material for: Changes in Resources and Volunteering: A Longitudinal Study of Active Engagement Among Older Europeans
Source: Gerontologist. 2024 May 20;65(1):gnae049. doi: 10.1093/geront/gnae049 (PMC11879309; doi:10.1093/geront/gnae049)
Supplement: gnae049_suppl_Supplementary_Materials [file gnae049_suppl_supplementary_materials.docx]

**Supplementary Material**

| **Supplementary Table 1**  *Distribution of Observations Across the Study Sample* | | | | | |  |
| --- | --- | --- | --- | --- | --- | --- |
|  |  |  |  |  |  |  |
| Participated in | | | | |  |  |
| Wave 4 | Wave 5 | Wave 6 | Wave 7 | Wave 8 | *N* | % |
|  |  |  |  |  | 6653 | 38.02 |
|  |  |  |  |  | 2689 | 15.37 |
|  |  |  |  |  | 1277 | 7.3 |
|  |  |  |  |  | 1254 | 7.17 |
|  |  |  |  |  | 985 | 5.63 |
|  |  |  |  |  | 761 | 4.35 |
|  |  |  |  |  | 624 | 3.57 |
|  |  |  |  |  | 551 | 3.15 |
|  |  |  |  |  | 513 | 2.93 |
|  |  |  |  |  | 424 | 2.42 |
|  |  |  |  |  | 374 | 2.14 |
|  |  |  |  |  | 285 | 1.63 |
|  |  |  |  |  | 250 | 1.43 |
|  |  |  |  |  | 186 | 1.06 |
|  |  |  |  |  | 183 | 1.05 |
|  |  |  |  |  | 137 | 0.78 |
|  |  |  |  |  | 59 | 0.34 |
|  |  |  |  |  | 56 | 0.32 |
|  |  |  |  |  | 44 | 0.25 |
|  |  |  |  |  | 44 | 0.25 |
|  |  |  |  |  | 40 | 0.23 |
|  |  |  |  |  | 36 | 0.21 |
|  |  |  |  |  | 27 | 0.15 |
|  |  |  |  |  | 21 | 0.12 |
|  |  |  |  |  | 18 | 0.1 |
|  |  |  |  |  | 7 | 0.04 |
|  |  |  |  |  | 17498 | 100 |

| **Supplementary Table 2**  *Descriptive Information of the Countries in the Study Sample* | | | | | | | |  |
| --- | --- | --- | --- | --- | --- | --- | --- | --- |
|  |  |  |  |  |  |  |  |  |
| Country | Social expenditures ^a^ | Volunteering (%) | | | | | Observations | Respondents |
|  |  | *0* | *1* | *2* | *3* | *4* |  |  |
| Luxembourg | 17983 | 38.2 | 12.8 | 18.4 | 21.2 | 9.5 | 1220 | 448 |
| Denmark | 15282 | 39.9 | 13.4 | 17.9 | 23.1 | 5.8 | 5455 | 1579 |
| Switzerland | 15279 | 42.5 | 10.0 | 19.2 | 22.0 | 6.3 | 4896 | 1329 |
| Sweden | 12012 | 48.7 | 11.1 | 17.3 | 17.9 | 4.9 | 3379 | 956 |
| Netherlands | 11846 | 33.5 | 9.6 | 16.1 | 30.4 | 10.4 | 2442 | 1042 |
| France | 10632 | 43.6 | 9.8 | 15.5 | 22.6 | 8.5 | 5630 | 1593 |
| Austria | 10499 | 48.3 | 12.7 | 16.8 | 18.3 | 4.0 | 4841 | 1388 |
| Germany | 10002 | 36.3 | 9.1 | 20.3 | 25.2 | 9.1 | 4112 | 1273 |
| Belgium | 9999 | 41.9 | 11.3 | 15.7 | 22.6 | 8.5 | 7044 | 2078 |
| Italy | 7607 | 51.3 | 9.4 | 12.1 | 18.9 | 8.4 | 3381 | 1054 |
| Spain | 5477 | 59.6 | 12.1 | 12.5 | 11.2 | 4.7 | 2502 | 750 |
| Greece | 4417 | 42.0 | 23.5 | 14.9 | 15.9 | 3.8 | 584 | 285 |
| Slovenia | 4338 | 53.6 | 11.1 | 15.0 | 12.7 | 7.5 | 2930 | 924 |
| Portugal | 4302 | 52.1 | 14.0 | 9.2 | 14.5 | 10.2 | 684 | 251 |
| Czech Republic | 3042 | 60.0 | 16.3 | 11.2 | 9.0 | 3.5 | 3454 | 974 |
| Croatia | 2380 | 45.4 | 9.5 | 14.6 | 19.4 | 11.1 | 315 | 156 |
| Poland | 2305 | 53.9 | 20.9 | 10.4 | 10.1 | 4.7 | 297 | 116 |
| Hungary | 2185 | 45.4 | 20.4 | 18.3 | 9.8 | 6.1 | 427 | 201 |
| Estonia | 2053 | 58.8 | 16.6 | 11.5 | 9.0 | 4.1 | 3817 | 1101 |
| Total |  | 46.4 | 12.0 | 15.8 | 19.2 | 6.7 | 57410 | 17498 |
| *Note*s. ^a^ Expenditures per inhabitant. Average over waves 4–8 at constant 2010 prices. | | | | | | |  |  |

| **Supplementary Table 3**  *Transitions in the Frequency of Volunteering (%)* | | | | | | |  |
| --- | --- | --- | --- | --- | --- | --- | --- |
|  |  | Time 2 | | | | | Total *n* |
|  |  | 0 | 1 | 2 | 3 | 4 |  |
| Time 1 | |  |  |  |  |  |  |
|  | 0 No volunteering | 49.3 | 15.2 | 14.9 | 15.7 | 4.9 | 100 |
|  | 1 Less than monthly | 62.4 | 14.0 | 13.0 | 8.9 | 1.7 | 100 |
|  | 2 Almost monthly | 46.3 | 10.6 | 19.9 | 19.9 | 3.3 | 100 |
|  | 3 Almost weekly | 38.2 | 5.3 | 17.0 | 28.8 | 10.8 | 100 |
|  | 4 Almost daily | 36.5 | 3.5 | 7.5 | 31.6 | 20.8 | 100 |

| **Supplementary Table 4**  *Transition in Resource Variables* | |  |  |
| --- | --- | --- | --- |
|  |  |  |  |
|  | Number of transitions over the waves | | |
|  | 0 | 1 | 2 |
| Change to worse health | 49900 | 7338 | 172 |
| Change to better health | 51183 | 6073 | 154 |
|  |  |  |  |
| Change to worse financial condition | 51819 | 5476 | 115 |
| Change to better financial condition | 50649 | 6624 | 137 |
|  |  |  |  |
| Transition to weekly work | 54992 | 2383 | 35 |
| Transition out of weekly work | 48329 | 8969 | 112 |
|  |  |  |  |
| Transition to weekly grandparenting | 52842 | 4528 | 40 |
| Transition out of weekly grandparenting | 52969 | 4387 | 54 |
|  |  |  |  |
| Transition to family care | 54802 | 2594 | 14 |
| Transition out of family care | 54952 | 2445 | 13 |
